# Methodology, outcome, safety and in vivo accuracy in traditional frame-based stereoelectroencephalography

**DOI:** 10.1007/s00701-017-3242-9

**Published:** 2017-07-05

**Authors:** Lars E. van der Loo, Olaf E. M. G. Schijns, Govert Hoogland, Albert J. Colon, G. Louis Wagner, Jim T. A. Dings, Pieter L. Kubben

**Affiliations:** 1 Department of Neurosurgery, Maastricht University Medical Center, Maastricht, Limburg The Netherlands; 2 School of Mental Health and Neuroscience, Maastricht University Medical Center, Maastricht, Limburg The Netherlands; 3 Academic Centre for Epileptology, Kempenhaeghe/Maastricht University Medical Centre, Heeze/Maastricht, Limburg The Netherlands

**Keywords:** Complications, Epilepsy surgery, In vivo accuracy, Stereoelectroencephalography, Stereotaxy

## Abstract

**Background:**

Stereoelectroencephalography (SEEG) is an established diagnostic technique for the localization of the epileptogenic zone in drug-resistant epilepsy. In vivo accuracy of SEEG electrode positioning is of paramount importance since higher accuracy may lead to more precise resective surgery, better seizure outcome and reduction of complications.

**Objective:**

To describe experiences with the SEEG technique in our comprehensive epilepsy center, to illustrate surgical methodology, to evaluate in vivo application accuracy and to consider the diagnostic yield of SEEG implantations.

**Methods:**

All patients who underwent SEEG implantations between September 2008 and April 2016 were analyzed. Planned electrode trajectories were compared with post-implantation trajectories after fusion of pre- and postoperative imaging. Quantitative analysis of deviation using Euclidean distance and directional errors was performed. Explanatory variables for electrode accuracy were analyzed using linear regression modeling. The surgical methodology, procedure-related complications and diagnostic yield were reported.

**Results:**

Seventy-six implantations were performed in 71 patients, and a total of 902 electrodes were implanted. Median entry and target point deviations were 1.54 mm and 2.93 mm. Several factors that predicted entry and target point accuracy were identified. The rate of major complications was 2.6%. SEEG led to surgical therapy of various modalities in 53 patients (69.7%).

**Conclusions:**

This study demonstrated that entry and target point localization errors can be predicted by linear regression models, which can aid in identification of high-risk electrode trajectories and further enhancement of accuracy. SEEG is a reliable technique, as demonstrated by the high accuracy of conventional frame-based implantation methodology and the good diagnostic yield.

## Introduction

Approximately 20% to 40% of epilepsy patients have seizures that are or become drug resistant [13, 23, 28, 36, 43, 63, 81]. In a significant part of these patients, surgery is superior to prolonged medical therapy [24, 84]. Successful surgery can lead to seizure freedom or significant seizure reduction and consequently lead to an improved quality of life and a lower morbidity/mortality rate [49, 57, 84].

The concept of the epileptogenic zone (EZ) is important for the presurgical approach in delineating the seizure focus and network accurately, resulting in an optimal, patient-tailored resection planning, which may lead to a better postoperative outcome [55, 61, 62]. The epileptogenic zone is defined as the “area of cortex that is indispensable for the generation of epileptic seizures,” and resection or disconnection of this zone is necessary for seizure freedom or reduction [47, 62].

Frequently, noninvasive diagnostic tools are adequate for the identification of the EZ. However, the number of highly complex patients in whom invasive intracranial diagnostic recordings are necessary has increased substantially the last years [41, 59, 60, 75]. Intracerebral depth electrodes can be used to detect the laterality of the EZ or the exact localization of seizure onset with a lower complication rate than registration with subdural strip and grid electrodes [1, 8, 10, 14, 16–20, 22, 29–34, 37, 42, 44, 46, 50, 52, 56, 67, 73, 74, 77, 78, 83, 85, 89]. In deep-seated areas such as the insular region, it is the only available method for long-term extraoperative intracranial recording. This technique of stereoelectroencephalography (SEEG) was originally developed by Talairach and Bancaud in Paris, France, over 50 years ago [3–6, 53, 68–72].

In the literature, four studies have assessed the in vivo accuracy of SEEG [2, 16, 30, 80], in contrast to the large number of studies analyzing the precision of different stereotactic procedures such as deep brain stimulation or biopsies [7, 9, 11, 21, 25, 27, 35, 38–40, 48, 54, 58, 65, 66, 76, 82]. Since higher accuracy may lead to improved EZ localization with consequently more precise resective surgery on the one hand and reduction of postoperative complications on the other, more data on the in vivo application accuracy of SEEG are of eminent interest.

In this longitudinal cohort study with prospectively collected data, we describe our experience with the SEEG technique since the start in our center in 2008. We discuss and evaluate our surgical methodology and in vivo application accuracy and consider the diagnostic and therapeutic yield and added value of the SEEG technique.

## Methods

### Patient selection

All patients with drug-resistant epilepsy and the suspicion of a focal onset who underwent stereotactic implantation of depth electrodes (SEEG) between September 2008 and April 2016 were included in this study. This research was not subject to the Medical Research Involving Human Subjects Act in The Netherlands (WMO in Dutch). All data were analyzed anonymously.

All patients underwent extensive preoperative evaluation, including video-electroencephalography (V-EEG), high-resolution (3-T) magnetic resonance imaging (MRI), neuropsychological examination and, on indication, positron emission tomography (PET), (inter)ictal single-photon emission computed tomography (SPECT) and/or magnetic encephalography (MEG). More recently, diffusion tensor imaging (DTI) and EEG-functional MRI (EEG-fMRI) have been added to the noninvasive presurgical workup.

All patients were discussed in our multidisciplinary patient management conference in the Academic Center for Epileptology (ACE). In a subset of these patients, frequently MRI-negative or with discongruent noninvasive test results, implantation of a subdural grid, strips or depth electrodes was indicated. SEEG was used if one or more of the following inclusion criteria were met: (1) a clear hypothesis about a deep-seated epileptogenic zone in one of the following cerebral regions: the mesial temporal lobe, interhemispheric regions, cingulate gyrus or the insular cortex; (2) congenital deep-seated lesions such as heterotopias or focal cortical dysplasia; (3) failure of previous noninvasive or invasive studies to clearly localize the EZ; (4) unknown lateralization; (5) multiple plausible hypotheses on the location of the EZ. Subdural grid and strip electrodes were chosen if there was a clear hypothesis about an epileptogenic zone closer to the cortical surface of the brain.

For the purpose of this study, we excluded patients who had undergone a bitemporal implantation (bilateral subdural strips and hippocampal depth electrodes).

### Image acquisition and trajectory planning

During the presurgical workup, a 3-T MRI scan for planning purposes was performed (Intera, Philips, Amsterdam, The Netherlands; T1-weighted rapid gradient echo sequence; 0.9–1.5 mm slice thickness; 0.5–1.0 mm squared pixel size; 256 × 256 or 512 × 512 matrix size) with gadopentate dimeglumine (Magnevist; Bayer Pharma AG, Berlin, Germany) at a dose of 0.2 ml/kg as a contrast agent to achieve optimal enhancement of vascular structures. Avoidance of vascular structures was obtained by means of gadolinium-enhanced MRI scan only, without digitalized angiography. Electrode planning for delineation of the suspected EZ and adjustment was performed on the basis of this MRI and the noninvasive seizure registration data by one of the staff epileptologists. Depending on the target localization, an orthogonal or oblique implantation trajectory was chosen. On the day of surgery, the Leksell frame (Elekta, Stockholm, Sweden) was placed under local anesthesia and a CT (SOMATO Definition Flash, Siemens, Munich, Germany; 1 mm slice thickness; 0.5–0.6 mm squared pixel s bize; 512 × 512 matrix size) or MRI scan (Intera, Philips, Amsterdam, The Netherlands; T1-weighted rapid gradient echo sequence; 1 mm slice thickness; 1.0 mm squared pixel size; 256 × 256 or 512 × 512 matrix size) with stereotaxy protocol was made. In the period 2008–August 2013 (*n* = 39 implantations), only a 3-T Leksell MRI was performed on the basis of which the electrode planning was done. After August 2013, stereotaxy CT was performed for all implantations (*n* = 37), as this significantly reduced the scanning time and therefore patient waiting time. Stereotactic MRI- or CT-imaging data were coregistered with the preimplantation MRI scan. Trajectory verification and adjustment, avoiding vascular structures, were performed by two neurosurgeons and an epileptologist (navigation software: StealthStation® FrameLink™, Medtronic Inc., Minneapolis, MN, USA (2008–January 2015, *n* = 61 implantations) or iPlan® Stereotaxy, BrainLAB AG, Feldkirchen, Germany (from March 2015 *n* = 15 implantations).

### Electrode implantation

**Fig. 1:**
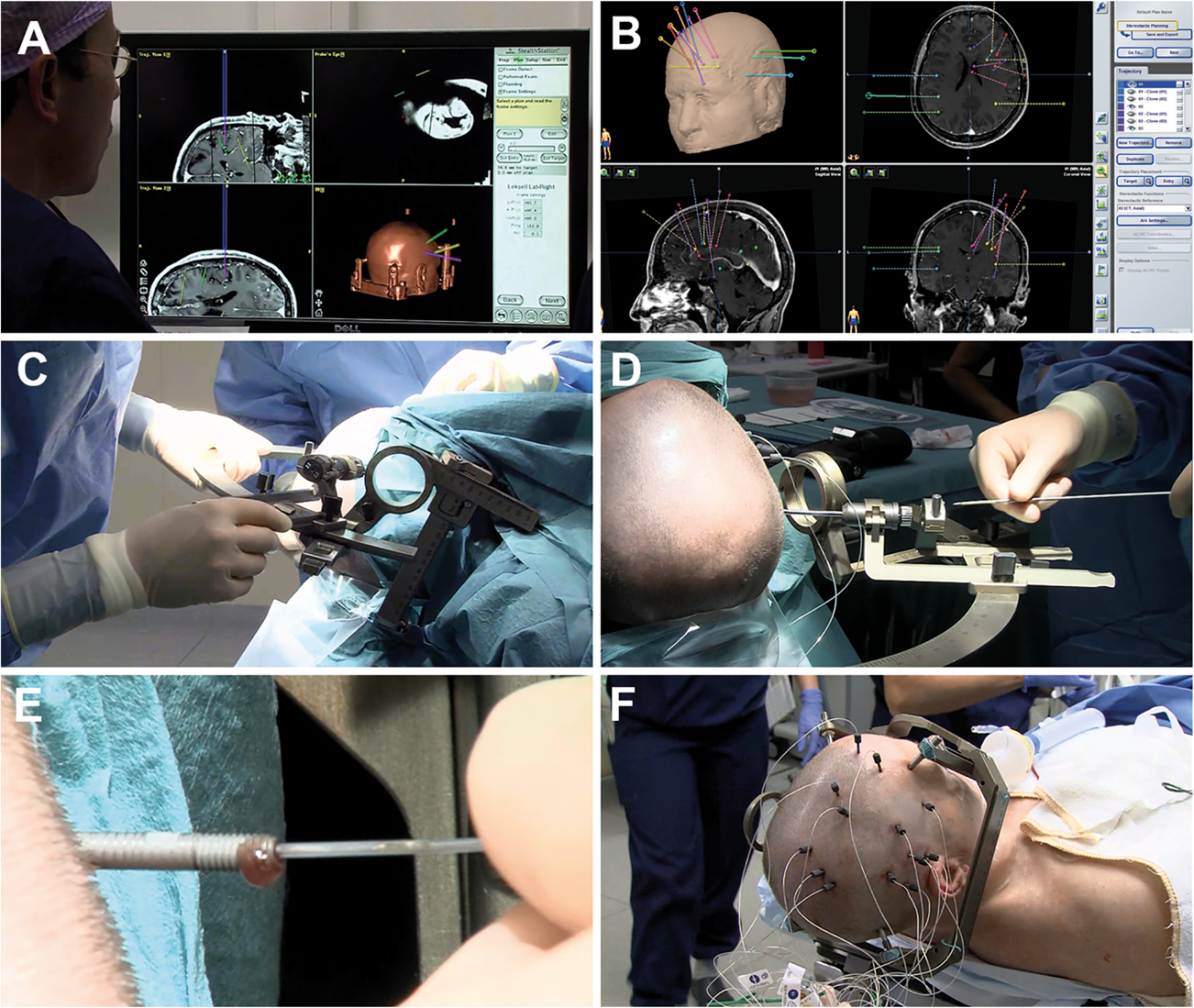
Methodology of electrode implantations. A and B: Planning of electrode trajectories using navigation software. C: Coordinates of the Leksell frame are set by the operating neurosurgeon. D: Introduction of the stylet through the guiding screw to a premeasured length to create the electrode tract. E: Insertion of the depth electrode through the screw. F: Aspect at the end of the procedure, after implantation of 13 depth electrodes and placement of fixation bolts

After general anesthesia, the patient with a Leksell frame was fixed in a head-holder (MAYFIELD®, Integra LifeSciences Corp., Plainsboro, NJ, USA). There was no strict policy to shave the hair of the head. After disinfection and draping, the coordinates for the first electrode were set by the operating neurosurgeon and reviewed by a second neurosurgeon or senior resident. A twist drill burr hole with a diameter of 1.2 mm was made with a handheld drill device (Colibri II, DePuy Synthes Power Tools, Palm Beach Gardens, FL, USA), and the dura was perforated using monopolar thermocoagulation (VIO 300D, Erbe Medical, Tübingen, Germany). The guiding screw (Dixi Medical, Beçanson, France) was fixated into the external table of the skull. Subsequently, the distance to the target was measured, and a stylet with this fixated distance (Dixi Medical, Beçanson, France, 0.8 mm diameter) was introduced through the brain parenchyma to create the straight target trajectory. Finally, the electrode (Microdeep® intracerebral electrodes; Dixi Medical, Beçanson, France) was inserted and fixated by tightening the bolt. The platinum/iridium electrodes have a diameter of 0.8 mm, a variable number of contacts (5–18), a contact length of 2 mm, and a 1.5-mm inter-contact distance. This procedure was repeated for each successive electrode. Various steps of the implantation process are visualized in Fig. 1.

### Postoperative phase

Within 24 h after implantation, CT and MRI scans, similar to the preoperative scanning protocols, were performed (1) to verify correct positioning of the electrodes and (2) to detect postoperative intracranial complications. Within 2 days following implantation, patients were transferred by ambulance to the Academic Center for Epileptology (ACE), location Kempenhaeghe, Heeze, The Netherlands, for a 1 to 4-week clinical period of seizure registration and for stimulation to map cortical functions. After registration stimulation, the patient was transferred back to ACE, location Maastricht University Medical Center (MUMC^+^), The Netherlands, and the depth electrodes were removed on the same day. The patient was observed 1 night and discharged the following day. The results of the registration and stimulation were discussed at the multidisciplinary patient management conference, and decisions for further treatment were made. When indicated, resective surgery was typically performed several months (average 4.5, range 1–15 months) after the SEEG implantation. In those patients not eligible for resection, deep brain stimulation (DBS) of the anterior nucleus of the thalamus or vagal nerve stimulation (VNS) was advised. After surgery, patients had a regular follow-up after 6 weeks, after 3 months and each year “on the birthday of the operation” to monitor seizure outcome and possible late-onset complications. Three months after surgery, a baseline 3-T MRI plus, in case of a temporal or parieto-occipital lobe resection, a control visual field analysis was performed.

### Data analysis

Analyzed data included the demographic and seizure characteristics, side, location, number and direction of implanted electrodes, and application accuracy. Additionally, procedure-related complications, target localization and the diagnostic outcome of SEEG were analyzed. We classified complications as major or minor; major complications included were mortality, urgent surgical reintervention after implantation or explantation, and persistent neurological deficits.

### Application accuracy

The intracranial position of implanted electrodes was assessed using postoperative CT with 1.0-mm-thick slices, which was automatically registered to the planning scan data with the same navigation software (iPlan® Stereotaxy, BrainLAB AG, Feldkirchen, Germany) used for trajectory planning (electrode trajectories that were planned using the StealthStation® FrameLink™ were also analyzed using the iPlan® software). Registration accuracy was verified by visual inspection of anatomical landmarks such as the lateral palpebral commissure and external acoustic meatus [86, 87].

**Fig. 2:**
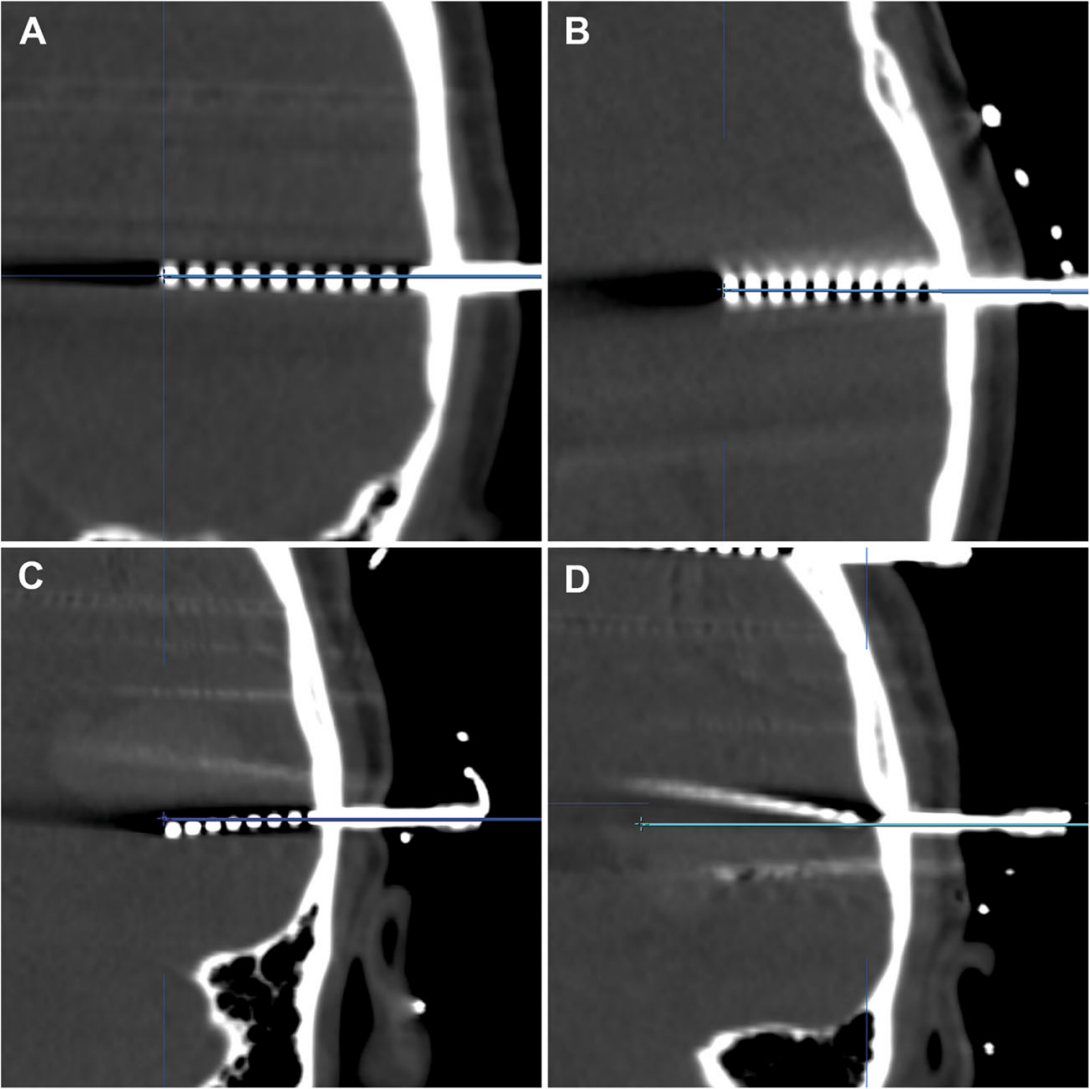
Three cases of in vivo application accuracy measurements on postoperative CT scans. The planned trajectories are shown as solid lines. For visualization purposes, the CT bone window setting was used (−200 to 800 HU). A: Coronal and (B) axial reconstructions of the same electrode, showing optimal positioning of the implanted electrode in comparison with the planned trajectory. Target point localization error (TPLE) was 0.83 mm for this electrode. C: Minor deviation in the coronal plane of an orthogonal electrode after insertion in the skull. The TPLE was 2.70 mm. D: Major deformation of the electrode in the coronal plane, with evidence of deviation in the other planes as well, resulting in a TPLE of 9.03 mm. TPLEs were measured in three different planes and calculated using the Euclidean distance

We measured the postoperative coordinates in coronal, axial and sagittal reconstructions by creating ad hoc trajectories for each of the implanted electrodes, using the ‘trajectory view’ to enable an accurate geometric visualization of the entry and target. The postoperative CT scan was windowed to the full range of Hounsfield units (HU; −1000 to 3000–4000), and the ‘bone setting’ (HU; −200 to 800) was used for verification. The center point of the hyperdensity of both the target and the entry was used for all measurements (Fig. 2).

The postoperative coordinates (x, y and z) of both the entry and the electrode tip (= target) were obtained from the fused data sets. These coordinates were compared with the expected, preoperative planning coordinates of the electrodes in all planes, and the directional errors in three directions (x: mediolateral, y: anterior-posterior, z: craniocaudal) were calculated.

**Fig. 3:**
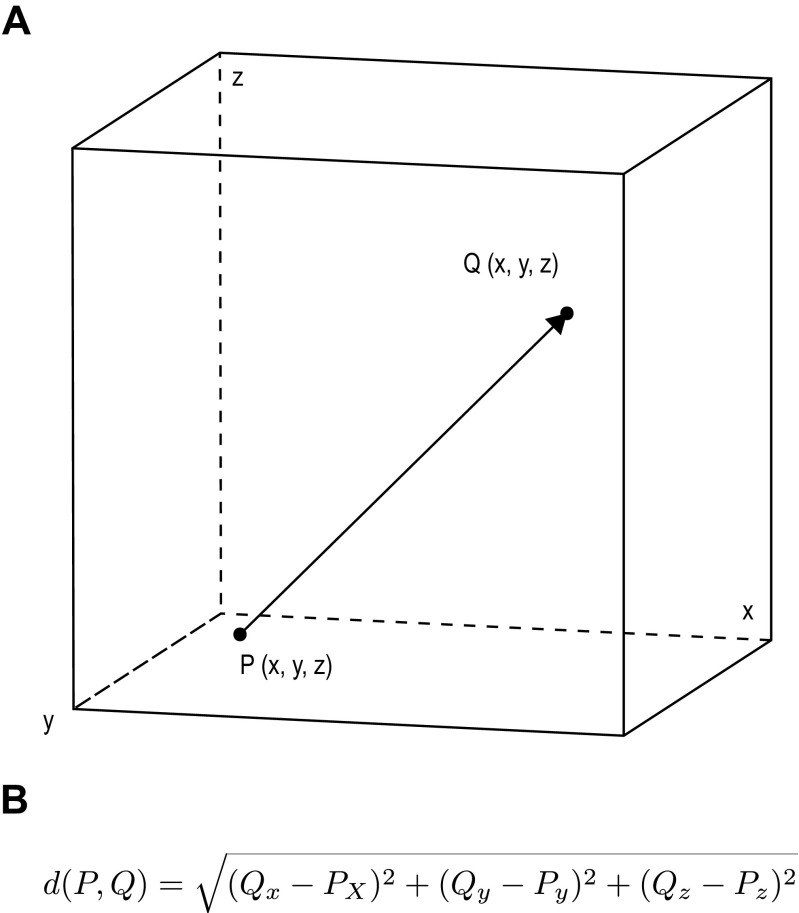
A: The concept of Euclidean distance. The tip of the planned trajectory is represented by point P, and the tip of the actual electrode is represented by point Q. The arrow is the Euclidean distance between both points. B: The Euclidean distance formula. For two points, the coordinates x, y and z are determined, and the Euclidean distance is defined as the square root of the sum of the squares of the difference between these coordinates

The entry and target point localization errors (EPLE/TPLE) were calculated for each electrode. EPLE was defined as the Euclidean distance between the planned entry coordinates and the post-implantation positions of the electrode entry point (Fig. 3A). TPLE was defined as the Euclidean distance between the planned target point and the tip of the electrode as visualized on postoperative imaging. Euclidean distances between two points in a three-dimensional space were calculated using the Euclidean distance equation (Fig. 3B).

A small Euclidean distance, thus a small localization error, indicates that a depth electrode is placed closely to the intended target, representing a high accuracy. Additionally, the directional errors were used to assess bias on the three individual planes to identify systematic errors in the stereotactic frame or the methods of measurement.

In addition to the localization errors, several explanatory variables (listed in the Results section) were measured. Linear models were used to investigate their predictive capabilities for EPLE and TPLE. Screw length decisions were based on skull thickness and skin-skull distance. Numbers of electrode contacts were chosen dependent on the intracerebral entry-target trajectory length. To reduce multicollinearity, these derivative variables were not included.

### Statistical analysis

Since the application accuracy data were not normally distributed, EPLE and TPLE were reported as median distances with their respective interquartile range. Initial bivariate analysis between explanatory variables and localization errors was performed with the Spearman correlation test for non-normally distributed numerical variables, and the Mann-Whitney U-test (in case of two independent variables) or Kruskal-Wallis H-test (for analysis of more than two independent variables) were used for categorical variables. Relevant explanatory variables were used for multivariate linear regression. Statistical significance was set at *p* < 0.05. Statistical analysis was performed with R 3.2.5 (R Foundation for Statistical Computing, Vienna, Austria). Geometric visualizations were constructed with Adobe Illustrator CC 2015 (Adobe Systems Incorp., San Jose, CA, USA).

## Results

### Patient demographics

Between September 2008 and April 2016, 71 patients underwent SEEG implantation. Three patients were implanted twice, and one patient was implanted three times leading to a total of 76 implantation procedures.

At implantation, the mean age of included patients was 31 years (age range 6–57 years); 35 patients (49.3%) were male. Seventeen of these were pediatric patients with a mean age of 12 years (age range 6–17 years). Mean epilepsy duration was 19 years (median 18 years, range 1–43 years). The seizure frequency ranged from multiple times a day to once a month. MRI abnormalities were present in 39 patients (54.9%) (Table 1).

**Table 1:** MRI abnormalities in patients who underwent SEEG implantations

MRI abnormality	Frequency (%)
None	32 (45.1)
Cortical dysplasia	15 (21.1)
Parenchymal defect	7 (9.9)
Mesiotemporal sclerosis	5 (7.0)
Heterotopia	5 (7.0)
Hippocampal sclerosis	3 (4.2)
Previous surgery	2 (2.8)
Cyst	1 (1.4)
Gliosis	1 (1.4)

### Implantation characteristics

A total of 902 depth electrodes were implanted, corresponding to an average of 12 electrodes per patient (range 3–22 electrodes). Data for in vivo application accuracy analysis were available for 866 electrodes. In another 36 electrodes (corresponding to 2 implantation sessions and 7 single electrodes from various implantations), the planning coordinates could not be identified because of absent trajectory data in patient records. Analysis of complications and diagnostic outcome was performed for all 76 implantations.

Localization of depth electrodes was unilateral in 35 procedures (46%) (22 were right and 13 were left hemispheric). Approximately one third of all electrodes (*n* = 272) were implanted orthogonally in relation to the sagittal plane; the other electrodes (*n* = 594) were placed in an oblique orientation.

The average duration of surgery was 136 min (median 124 min, range 66–290 min). Bilateral implantations took significantly longer than unilateral procedures (mean operative times were 158 versus 132 min, respectively; *p* = 0.047).

### Application accuracy

#### Euclidean distance

The median EPLE was 1.54 mm [interquartile range (IQR) 0.92–2.28 mm], and the median TPLE was 2.93 mm (IQR 1.98–4.20 mm). Maximum EPLE and TPLE were 26.55 and 45.76 mm, respectively, caused by slipping of the drill and extreme deviation of one electrode. EPLE was <2 mm in 67.3%, and TPLE was <2 mm in 25.8%. Approximately 29.8% of EPLEs and 57.3% of TPLEs showed a localization error between 2 and 5 mm, and in 2.9% of EPLEs and 17.0% of TPLEs the errors were more than 5 mm.

#### Directional errors

**Fig. 4:**
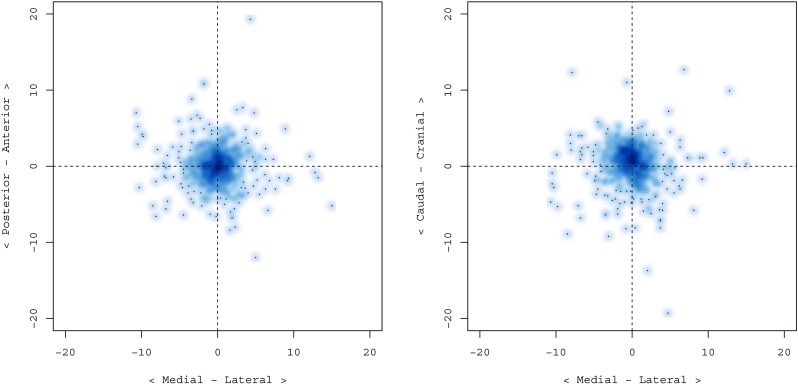
Density scatterplots of electrode target directional errors. Higher density areas represent more electrodes with the same directional errors. In the left pane, the directional errors in the medial-lateral X-direction (horizontal axis) and the anterior-posterior Y-direction (vertical axis) are shown. The right side plot shows directional errors in the medial-lateral X-direction (horizontal axis) and the caudal-cranial Z-direction (vertical axis). The graphs illustrate a small deviation in the lateral and cranial directions

Median entry directional errors in the mediolateral (X), anterior-posterior (Y) and Z (craniocaudal) direction were 0.00 (IQR -0.73–0.63), 0.50 (IQR -0.20–1.30) and 0.00 (IQR -0.40–1.20), respectively, indicating a small entry displacement in the anterior direction. X, Y and Z directional errors for the target were −0.10 (IQR -1.40–1.18), 0.00 (IQR -1.30–1.20) and 0.70 (−0.70–1.90), respectively, demonstrating minor deviation in the lateral and cranial directions (Fig. 4).

#### Explanatory variables

Analysis of explanatory variables was performed using bivariate analysis (Tables 2 and 3). In summary, the type of electrode, skin-skull distance, skull angle, screw length, intracranial electrode bending and quality of image registration were associated with localization errors (both EPLE and TPLE). Intracranial electrode length, planning scan modality (MRI/CT) and temporo-polar localization demonstrated a significant relationship with TPLE (electrodes implanted in the temporal pole had smaller localization errors). There was no statistically significant difference between the various surgeons performing the operation. Linear models for EPLE and TPLE were created using all relevant explanatory variables (Table 4).

**Table 2:** Bivariate analysis of numerical variables and localization errors ^*a*^

Variable	Median (IQR)	EPLE		TPLE	
		Test	P	Test	P
Age (years)	30 (20–40)	−0.027	0.423	0.045	0.187
Skin-skull distance (mm)	6.4 (5.1–8.1)	0.161	<0.001 ^*b*^	0.258	<0.001 ^*b*^
Skull thickness (mm)	6.7 (4.9–8.7)	0.079	0.020 ^*b*^	0.170	<0.001 ^*b*^
Acute skull angle (degrees)	76.1 (66.5–84.2)	−0.273	<0.001 ^*b*^	−0.262	<0.001 ^*b*^
Intracranial length (mm)	41.0 (30.7–55.5)	-	-	0.079	0.020 ^*b*^

^a^EPLE, entry point localization error; TPLE, target point localization error. None of the values are normally distributed (Shapiro-Wilk normality test *p*-values are < 0.001 for all variables). Bivariate analysis (Spearman correlation) was performed. The relationship between EPLE and intracranial electrode length is not relevant

^b^Significant

**Table 3:** Bivariate analysis of categorical variables and localization errors ^*a*^

Variable	Categories	Frequency (%)	EPLE		TPLE	
			Median	P	Median	P
Planning scan modality	MRI	404 (46.7)	1.53	0.351	3.01	0.035 ^*b*^
	CT	462 (53.3)	1.57		2.80	
Planning software	Medtronic	682 (78.8)	1.54	0.463	2.93	0.594
	Brainlab	184 (21.2)	1.54		2.96	
Electrode direction	Oblique	594 (68.6)	1.50	0.023 ^*b*^	2.89	0.029 ^*b*^
	Orthogonal	272 (31.4)	1.66		3.01	
Electrode contacts	5	52 (6.4)	1.63	0.856	2.63	0.433
	8	173 (21.4)	1.48		2.70	
	10	148 (18.3)	1.56		2.82	
	12	137 (16.9)	1.50		2.90	
	15	122 (15.1)	1.61		3.13	
	18	178 (22.0)	1.52		3.12	
Surgeon	A (S)	239 (27.6)	1.61	0.517	2.62	0.128
	B (D)	59 (6.8)	1.46		2.77	
	C (K)	50 (5.8)	1.61		3.29	
	D (S + V)	225 (26.0)	1.48		2.85	
	E (D + V)	8 (0.9)	0.90		2.51	
	A + C (S + K)	75 (8.7)	1.68		3.00	
	B + C (D + K)	91 (10.5)	1.52		3.30	
	A + B (D + S)	119 (13.7)	1.61		3.00	
Temporal pole	Yes	124 (14.3)	1.48	0.063	2.71	0.002 ^*b*^
	No	742 (85.7)	1.56		3.00	
Screw length	20 mm	36 (11.0)	1.24	0.003 ^*b*^	2.51	0.018 ^*b*^
	25 mm	223 (68.4)	1.57		3.14	
	30 mm	55 (16.9)	1.91		3.20	
	35 mm	12 (3.7)	3.26		4.13	
Intracranial bending	No	771 (89.0)	1.52	<0.001 ^*b*^	2.74	<0.001 ^*b*^
	Yes	95 (11.0)	1.97		5.65	
Quality of registration	Optimal	777 (89.7)	1.52	0.001 ^*b*^	2.77	<0.001 ^*b*^
	Suboptimal	83 (9.6)	2.01		4.17	
	Bad	6 (0.7)	1.94		5.13	

^a^EPLE, entry point localization error; TPLE, target point localization error; MRI, magnetic resonance imaging; CT, computed tomography. None of the variables are normally distributed (Shapiro-Wilk normality test *p*-values are all < 0.001). A Kruskal-Wallis H-test or Mann-Whitney U-test was performed for all variables

^b^Significant

**Table 4:** Multivariate analysis ^*a*^

Variable	Reference	Compare	Coefficient	SE	P
Outcome = EPLE					
Intercept			3.666	0.433	<0.001
Electrode direction	Oblique	Orthogonal	0.454	0.129	<0.001
Temporal pole	No	Yes	0.435	0.178	0.015
Planning scan modality	CT	MRI	−0.330	0.110	0.003
Skin-skull distance			0.088	0.025	<0.001
Skull angle (acute)			−0.036	0.005	<0.001
Outcome = TPLE					
Intercept			1.581	0.224	<0.001
Intracranial bending	No	Yes	3.553	0.265	<0.001
EPLE			0.523	0.051	<0.001
Skull thickness			0.089	0.028	0.001

^a^EPLE, entry point localization error; TPLE target point localization error; SE, standard error

### Complications

Five patients (6.6%) developed intracranial hematomas, all but one related to the entry point of one or more electrodes (Table 5). Three of these patients were asymptomatic, and one had mild transient neurological deficits (right arm paresis), and no interventions or changes in treatment were needed. These complications were considered minor. One patient developed an acute intracerebral hematoma requiring emergency craniotomy for evacuation of the hematoma and removal of all depth electrodes, resulting in significant clinical improvement of the presenting hemiparesis and dysphasia. Two patients developed right arm paresis (MRC-grade 4 and 3, respectively) without evidence of intracranial hematoma on CT imaging. Both events were related to an intended electrode entry site adjacent to the motor cortex and local edema formation. One of these patients showed complete remission of clinical symptoms; the other showed significant improvement after rehabilitation therapy. There were two surgical complications. In one patient, there was a malposition of one fixation screw through the tabula externa and interna into the cranial cavity. Because the patient was asymptomatic and the risk of surgery to remove the screw was considered too high, the screw was left in situ. No clinical or radiological complications were detected during a follow-up of 12 months. In another implantation session, the drill broke, without any adverse consequences or the requirement of additional surgical intervention. Total rates for minor complications were 9.2% (*n* = 7) and 2.6% (*n* = 2) for major complications. Regarding the total number of implanted electrodes (*n* = 902), the risk of a major complication per electrode was 0.22%, assuming that a single electrode is responsible for each hemorrhagic complication.

**Table 5:** Complications

Complication	Classification	Total, N
Hemorrhagic		
Intracerebral hematoma (persistent paresis and dysphasia)	Major	1
Subdural hematoma [1 transient arm paresis (MRC 4), 2 asymptomatic]	Minor	3
Minor subarachnoid bleeding (asymptomatic)	Minor	1
Non-hemorrhagic		
Right upper limb paresis (MRC 3, persistent)	Major	1
Right hand paresis (MRC 4, transient)	Minor	1
Surgical		
Screw malposition (asymptomatic)	Minor	1
Broken drill (asymptomatic)	Minor	1

### Diagnostic value

The mean duration of seizure registration was 16 days (range 6–31 days). In the analyzed time period, SEEG implantation led to surgical therapy of various modalities in 53 patients (69.7%). The mean follow-up period after therapeutic interventions was 15 months (range 1–84 months).

An overview of treatment strategies after SEEG is presented in Table 6. The epileptogenic zone (EZ), responsible for the seizures and eligible for resection, could be localized in 38 implantation sessions (50%). In total, 33 resective operations (43.4%) were performed. One operation was planned at the time of this research after recent registration (for a total of 44.7% resections and planned resections). Resective surgery was much more likely in patients who demonstrated MRI abnormalities (χ^2^ = 30.136, *p* < 0.001).

**Table 6:** Therapeutic modalities following SEEG procedures ^a^

Outcome	Frequency (%)
Surgical therapy	
Resection	
* Performed*	33 (43.4)
* Planned*	1 (1.3)
Vagal nerve stimulation	
* Performed*	11 (14.4)
* Planned*	2 (2.6)
Deep brain stimulation	
* Performed*	5 (6.6)
* Planned*	2 (2.6)
Thermocoagulation	4 (5.3)
No surgical therapy	
No surgical treatment (various reasons)	7 (9.2)
Proposition not yet known	7 (9.2)
Additional implantation necessary	4 (5.3)

^a^VNS, vagal nerve stimulation; DBS, deep brain stimulation

In 27 cases (35.5%), patients were not identified as resection candidates because of sampling errors, multifocal or bilateral EZs, or the presence of an EZ in an eloquent cortical area. Of these patients, 11 underwent VNS implantation, with another 2 patients scheduled for VNS implantation in the following 2 months (total of 17.1%). Five patients were treated with anterior thalamic DBS, and two patients had DBS procedures scheduled in the near future (total of 9.2%). Seven patients (9.2%) received no surgical treatment. Of these, one patient died during the period between the SEEG and the proposed resection. This death was attributed to sudden unexpected death in epilepsy (SUDEP). Two patients who were eligible for resection refused surgery. In one patient, the clinical condition did not allow for surgery. Three patients had multifocal EZs and were net eligible for VNS or DBS.

Four patients (5.3%) were treated with SEEG-guided thermocoagulation of the EZ (based in a periventricular heterotopia). In seven patients (9.2%), SEEG implantations were performed very recently, and therefore no resection proposition had been formulated at the time of this study. In four cases (5.3%), additional implantations were necessary. No patients were lost to follow-up.

## Discussion

### Key results: In vivo application accuracy

We present the largest in vivo application analysis of conventional frame-based SEEG electrode implantation published to date. The number of quantified trajectories is on the same order of magnitude as a comparison between conventional methodology and robot-assisted surgery by Cardinale et al. [16] and an analysis of robot-assisted implantations by González and colleagues [30].

Four studies describe the in vivo accuracy of SEEG (Table 7), but only two of them assess a conventional frame-based methodology. Cardinale et al. [16] reported a median Euclidean entry error of 1.43 mm and a median target error of 2.69 mm (517 electrodes). Balanescu et al. [2] developed a customized, disposable stereotactic fixation device and reported a mean TPLE of 1.64 mm. They calculated entry deviation in a different way, using the normal distance between the entry point and the trajectory line. Since Euclidean distance is the sum of the error in three directions, it is always larger than the normal error distance, making it difficult to directly compare this value with our results and the results of other studies. Despite their analysis consisting of only 52 trajectories, the high accuracy of their custom-made fixation device has to be acknowledged.

**Table 7:** In vivo localization errors in clinical studies using various stereotactic systems^a^

Study	Stereotactic system	Trajectories	EPLE (MM)	TPLE (MM)
Cardinale et al., 2013 [16]	Talairach (frame based)	517	1.43	2.69
	Neuromate (robot)	1050	0.78	1.77
Balanescu et al., 2014 [3]	StarFix (frame based)	52	0.68 ^b^	1.64 ^b^
González et al., 2015 [31]	ROSA (robot)	500	1.2	1.7
Verburg et al., 2016 [78]	VarioGuide (frameless)	89	-	3.5
Present study, 2016	Leksell (frame based)	854	1.54	2.93

^a^EPLE, entry point localization error; TPLE target point localization error. Median errors are shown

^b^Mean errors. EPLE was not calculated as Euclidean distance, but as normal distance

Cardinale et al. [16] were also the first to demonstrate that robot-assisted SEEG implantations have a superior in vivo accuracy in comparison with other methods, with a median EPLE of 0.78 mm and a median TPLE of 1.77 mm. These results were confirmed by González et al. [30], who reported median values of 1.2 mm and 1.7 mm for EPLE and TPLE, respectively. Verburg et al. [80] analyzed the in vivo application accuracy of a frameless stereotactic implantation methodology using laser surface matching and skin fiducial markers. The median TPLE in their study was 3.5 mm, although the number of verified trajectories was much lower.

All of the above studies used automatic registration of postoperative MRI or CT to presurgical imaging to assess the position of depth electrodes, although there were minor dissimilarities in scanning protocols and timing of imaging. Cardinale et al. [16] used intraoperative postimplantation O-arm 3D–CT imaging, whereas the other three studies performed postoperative scans. They mentioned that the use of an intraoperative MRI or CT scan for electrode localization is potentially beneficial, as immediate repositioning can be performed if suboptimal placement is detected. However, they do not report how often this was necessary. Since hemorrhagic complications could already have occurred after suboptimal placement, the added value of intraoperative imaging and electrode repositioning is not clear.

MRI and CT are the most commonly used imaging modalities for the postoperative accuracy analysis of electrode location [45, 76]. Both MRI and CT are described as being equally accurate for postoperative localization of different electrode types, including DBS and SEEG electrodes [45, 58, 76, 79]. MRI is more susceptible to image distortion compared to CT, but MRI has no radiation exposure, and images can be obtained at higher spatial resolution. CT offers sharp and high-contrast electrode tip visualization, facilitating deviation analysis [26, 58].

As proposed by Bot et al. [11], directional errors can provide additional information about stereotactic accuracy. They can be used to assess systematic errors of the stereotactic system in one of the three planes. In our study, directional errors showed minor target deviation in the lateral and cranial directions. No systematic error in the anterior or posterior direction was observed. Taking the interquartile range of these errors into account, we think the systematic error in our stereotactic frame is negligible.

Cardinale et al. [16] were the first, and to date the only ones, to demonstrate the association between localization errors and several explanatory variables. In our study, an analysis of explanatory variables following their protocol showed that variables associated with EPLE were the skin-skull distance and angle at the skull (thicker tissue and more oblique angles make drilling less precise because of possible drill bending), type of electrode (orthogonal versus oblique), planning scan modality (MRI demonstrated less precision, possibly because of registration inaccuracy) and temporal localization. As a consequence of the inward curvature of the temporal skull, deviation of the drill tip is probable in case of oblique electrode positioning. Variables associated with TPLE were EPLE and all variables associated with it, intracranial electrode bending and skull thickness. The length of the electrode was not significantly associated in multivariate analysis, corresponding with our observation that short electrodes can show bending immediately after entry in the skull, resulting in a large TPLE. This contrasts with the findings of Cardinale et al. [16] that a longer intracranial trajectory is associated with a larger TPLE.

In contrast with other stereotactic (implantation) techniques, such as deep brain stimulation or tumor biopsies, SEEG trajectories and insertion angles are much more variable. DBS leads are usually placed in a comparable direction because of the close proximity of the different anatomical target areas. SEEG electrodes are more variable regarding the insertion length (varying between 5 and 18 electrode contact points), insertion angle (including different stereotactic techniques for oblique and orthogonal electrodes) and insertion location. Also, trajectories cannot always avoid passing through the lateral ventricle, which may contribute to decreased accuracy. Electrodes are inserted through twist burr holes, in contrast to larger burr holes with a visual inspection of possible electrode deviation at the cortical surface in a DBS procedure, for example. These differences underline the importance of explanatory variable analysis and the detection of high-risk trajectories.

Determination of postoperative coordinates was ambiguous for some of the electrodes with the largest EPLE and/or TPLE. In certain electrodes (*n* = 11), the coordinates as obtained from patient files appeared to be incorrect, presumably because of errors in the operative reports. Ten of these 11 electrodes were implanted 2010. We decided to include these electrodes nonetheless, since inaccuracies in postoperative reports are a factor to acknowledge during retrospective analysis of SEEG accuracy. Because of non-normality and use of median values, this subset of electrodes likely has a very limited impact on the primary outcome measures.

### Complications

In the past, several studies demonstrated the safety of SEEG and the lower rate of complications in comparison with implantation of subdural grids or strips. Recently, Mullin et al. [51] performed a comprehensive systematic review of SEEG-associated complications. They identified 121 surgical complications in 30 studies from 1983 to 2014, corresponding to a pooled prevalence of 1.3% (95% CI 0.9–1.7%). The most common complications were intracranial hemorrhages [pooled prevalence 1.0% (95% CI 0.6 1.4%)], followed by infections (0.8%, 95% CI 0.31–1.2%). Technical complications presented in 0.6% (95% CI -0.1–1.4%) of implantations. González et al. [30] reported a total complication rate of 4.0% in 100 implantations (major complications in 1.0%, minor complications in 3.0%). This study was not included in the meta-analysis, but overlaps with earlier research [32]. Recent research by Yang et al. [88] shows a global complication rate of 16.7%, with a complication rate for hemorrhage and infection of 4.2%. A very recent study by Bourdillon et al. [12] reports a major complication rate of 1.52% and a minor complication rate of 2.09%. In their large patient cohort, they demonstrated that insular trajectories do not have a higher risk of intraparenchymal hematoma.

We had two (2.6%) major complications, both resulting in persistent neurological deficits. In addition, we reported seven (9.2%) minor complications (4 hemorrhagic without clinical symptoms, 2 technical and 1 transient neurological deficit attributable to postoperative edema formation). We did not observe an association between the magnitude of localization errors and the occurrence of complications. Our global complication rate is higher than reported in the systematic review, although Mullin et al. [51] advocate for careful interpretation of their results because of presumed underreporting of minor complications. Moreover, they discovered that centers reporting smaller sample sizes also reported higher complication rates [51].

For direct comparison of complication rates, a subgroup of implantations performed with the traditional Talairach frame by Cardinale et al. [16] seems to be the most suitable, since the implantation method is highly comparable. They reported a global complication rate of 4.5% and a major complication rate of 2.6%, however noting that the global complication rate is likely an underrepresentation of the actual rate. Their robot-assisted implantation complication rate is much lower, and presumably even lower than the rate reported [15].

### Therapeutic consequences

After SEEG registration, 69.7% of all implanted patients underwent any modality of surgical therapy. Our surgical resection rate of 43.4% was somewhat lower than in comparable studies [16, 17, 29, 31, 50, 64], possibly because there were relatively many patients who demonstrated no MRI abnormalities. Over 20% of all patients underwent VNS or DBS therapy, which is less frequently described in the literature. Four patients underwent thermocoagulation of a periventricular heterotopy, a technique introduced in our center in 2016.

### Strengths and limitations

The main strengths of this study are the largest number of frame-based implanted SEEG electrodes to date and the robust methods of measurement, using both the Euclidean-distance method and three-dimensional analysis using directional errors. Reproducibility is likely high, provided that indication, trajectory planning and surgical methodology are performed under similar conditions. Most centers use conventional frame-based methods for SEEG, and a direct comparison of the results should be possible. As argued, SEEG implantation differs significantly from various other stereotactic approaches. Since only a few studies report in vivo accuracy data, our research is helpful for better understanding of SEEG accuracy and determination of the best SEEG approach.

The main limitations of our study are the retrospective nature of the research and the image registration phase, because multimodality fusion unavoidably introduces a small error. Because of the millimeter scale of localization errors, this error has to be taken into consideration. As discussed earlier, robot-assisted implantations have a superior accuracy to frame-based SEEG implantations. This small difference in accuracy does not seem to influence the complication rate, but is a very important factor to acknowledge.

### Suggestions for further research

The methods used for measurements of the entry and target point coordinates are clearly defined. Nonetheless, the obtained values for TPLE and EPLE could vary among several researchers, which could be assessed by calculating intra- and interobserver agreement in electrode localization.

In the present study, several statistically significant explanatory variables were assessed. More comprehensive mathematical prediction models for SEEG accuracy based on these variables can be constructed in the future using machine learning techniques. These models can be used for identification of high-risk electrodes and aid in determining optimal trajectories. Because of the many electrodes that need to be analyzed, the development of a reliable software tool that can automatically detect the postoperative entry and target points can be extremely beneficial to speed up analysis and aid mathematical modeling.

As argued before, the accuracy of multimodality image registration has a direct influence on the measurement of localization errors in stereotaxy. Improvement of this accuracy using optimization of image registration tools is a topic for future discussion. As advocated by Cardinale et al. [16], research-only software that is not yet certified for clinical use should not be ignored and can possibly play a role in optimizing multimodality fusion. Obviously such tools should be validated before clinical implementation.

## Conclusion

In this study, we demonstrated that the in vivo application accuracy of conventional frame-based SEEG implantations (*n* = 866 electrodes) is 1.54 mm and 2.93 mm for electrode entries and targets, respectively. These results are comparable with the experience of other epilepsy surgery centers described in the literature. Additionally, we demonstrated that entry and target point localization errors can be predicted by linear regression models. Explanatory variables that predict entry point localization error are electrode direction (orthogonal/oblique), temporal implantations, planning scan modalities (CT/MRI), skin-skull distance and skull angle. Target point localization error can be predicted by entry point localization error and all variables related to it, electrode bending and skull thickness. The traditional frame-based methodology is safe and the diagnostic yield satisfactory, although higher accuracy and safety may be obtained in robot-assisted stereotactic neurosurgery.
